# The effect of DNA degradation bias in passive sampling devices on metabarcoding studies of arthropod communities and their associated microbiota

**DOI:** 10.1371/journal.pone.0189188

**Published:** 2018-01-05

**Authors:** Henrik Krehenwinkel, Marisa Fong, Susan Kennedy, Edward Greg Huang, Suzuki Noriyuki, Luis Cayetano, Rosemary Gillespie

**Affiliations:** 1 Department of Environmental Sciences, Policy and Management, University of California, Mulford Hall, Berkeley, California, United States of America; 2 Center for Comparative Genomics, California Academy of Sciences, San Francisco, California, United States of America; 3 Center for Geo-Environmental Science, Rissho University, Saitama, Japan; Scientific Research Centre of the Slovenian Academy of Sciences and Art, SLOVENIA

## Abstract

PCR amplification bias is a well-known problem in metagenomic analysis of arthropod communities. In contrast, variation of DNA degradation rates is a largely neglected source of bias. Differential degradation of DNA molecules could cause underrepresentation of taxa in a community sequencing sample. Arthropods are often collected by passive sampling devices, like malaise traps. Specimens in such a trap are exposed to varying periods of suboptimal storage and possibly different rates of DNA degradation. Degradation bias could thus be a significant issue, skewing diversity estimates. Here, we estimate the effect of differential DNA degradation on the recovery of community diversity of Hawaiian arthropods and their associated microbiota. We use a simple DNA size selection protocol to test for degradation bias in mock communities, as well as passively collected samples from actual Malaise traps. We compare the effect of DNA degradation to that of varying PCR conditions, including primer choice, annealing temperature and cycle number. Our results show that DNA degradation does indeed bias community analyses. However, the effect of this bias is of minor importance compared to that induced by changes in PCR conditions. Analyses of the macro and microbiome from passively collected arthropod samples are thus well worth pursuing.

## Introduction

Metabarcoding and metagenomics are currently developing into routine applications for arthropod community ecology [1–2]. The sequencing of molecular barcodes from community samples promises rapid and unprecedented insights into the biodiversity of whole ecosystems [3–8]. Amplicon sequencing even makes it possible to analyze arthropod communities and their associated microbiota in parallel [9]. However, several taxonomic biases have been identified, which can lead to flawed diversity estimates in metabarcoding. Particular attention has been given to PCR amplification bias, which can skew community sequencing data. The exponential amplification of template DNA can greatly exaggerate even small differences in PCR efficiency between taxa. Several experimental approaches have been suggested to mitigate amplification bias, including selection of degenerate primers [10], barcode markers with conserved priming site [11–14] and reduction of PCR cycle number [15]. Recent research also suggests metagenomic protocols to avoid PCR altogether [16–17]. However, aside from PCR bias, other factors could affect the qualitative and quantitative efficiency of metabarcoding studies.

One bias in particular that has been largely neglected, concerns differential DNA degradation. The current study focuses on this bias. Differential DNA degradation could affect arthropod community samples and their associated microbiome in two ways. First, DNA could degrade at different rates in different taxa [18], leading to over or underrepresentation of species in a community sample. For example, taxa with different body sizes can show different rates of DNA degradation during long term storage [19]. And secondly, arthropod community samples are often subjected to long periods of suboptimal storage conditions due to being collected with passive sampling methods. Most passive sampling devices, e.g. Malaise or pitfall traps, are left in the field for several weeks, and thus expose specimens to varying times of suboptimal storage. Such conditions could amplify degradation bias in comparison to samples that are immediately frozen after collection [20]. Also, different specimens will enter the trap at different times and thus could show different rates of DNA degradation. Consequently, qualitative and quantitative metabarcoding results from passive sampling devices could be distorted, even in the absence of PCR amplification bias. Considering that many large international projects on biodiversity exploration rely on passive sampling devices [21], degradation bias could be a significant obstacle. The importance and magnitude of this degradation bias remains to be tested.

A straightforward approach to test for degradation bias would be the comparative analysis of species compositions from degraded and non-degraded DNA of the same community sample. Degradation bias should lead to skewed proportions of high and low molecular weight DNA between species: If one taxon is more degraded than another, the degraded taxon will be overrepresented in low molecular weight and underrepresented in high molecular weight DNA. In theory, this association could be simply tested using PCR primers that target short and long DNA fragments. However, PCR amplification bias between the primer pairs could completely override the effect of degradation bias, making it impossible to detect. Alternatively, PCR free libraries with different insert sizes could be used to study the taxon composition of intact and degraded DNA. But compared to a PCR based approach, this will lead to a considerable increase of workload and cost. A simple solution to this problem is to separate different DNA size fractions of the same sample. By performing PCR using the same primer pair on the high and low molecular weight fractions, amplification bias is excluded. This approach should provide a simple means to estimate, if the DNA of certain taxa in a community sample degraded faster than that of others.

Here we use a simple DNA size selection protocol to test for degradation bias in arthropod community samples and their associated microbiota from native rainforests on the Hawaiian Archipelago. We analyze mock community samples containing various species of arthropods belonging to 14 orders. The samples were either immediately frozen after collection, or stored at room temperature. The warm temperature treatment was meant to mimic conditions in a passive sampling device. To test degradation under field conditions, we additionally analyzed the content of two Malaise traps. After DNA extraction, we separated degraded from non-degraded DNA for each sample. Each fraction was then amplified for mitochondrial COI to target arthropods and ribosomal 16SrDNA to target the associated microbiome. By comparing the taxon composition of degraded (low molecular weight) and non-degraded (high molecular weight) fractions, we quantified degradation bias. We compared the recovered degradation bias with PCR bias in a second experiment, by evaluating the effects of different PCR conditions on taxon recovery. We tested for an effect of exact PCR replicates of the same sample, a reduction of PCR cycle numbers, an increase of PCR annealing temperature and the use of two different marker sets on taxon recovery.

## Materials and methods

### Estimation of DNA degradation bias from mock communities

Two samples of several hundred arthropod specimens from 14 orders were collected in spring 2016 by beating vegetation in native rainforests of Hawaii Island (Puumakaala Forest Reserve, Lat: 19.620° Lon: -155.416°). Collecting permits were issued by the Hawaii Department of Land and Natural Resources in Honolulu. No protected species were collected. All specimens were stored in 99% ethanol. One sample was immediately frozen at -20°C and kept continuously frozen for exactly 4 weeks. The other was kept at room temperature for the same period. These two treatments were supposed to mimic **1.** a field collected sample that is immediately frozen after collection and **2.** a passive sampling device, in which the sample is exposed to unfavorable conditions for DNA preservation. Passive sampling devices can be left in the field for varying times, from few days to several months. With 4 weeks, we chose an intermediate time, comparable to many actual field studies and at the same time exposing samples to unfavorable conditions for a time sufficient to induce DNA degradation. After 4 weeks, specimens were sorted to species where possible, or to morphotypes that likely correspond to different species, and were dried on Kimwipes. Fifteen mock communities were prepared for each treatment (freezer and room temperature). Specimens were pooled in 2 ml Eppendorf tubes, using randomized amounts of tissue from different taxa. We aimed to make communities as similar as possible and added the same species into different communities were possible. For few taxa, we could not make exact replicates, because of limited numbers of specimens available and had to use close relatives for different communities. These community samples contained between 5.25–24.12 mg (mean = 15.36 mg) of tissue per pool. While entire bodies of small specimens were used, large specimens were cut into pieces with sterile razor blades. The dry weight of each specimen in the communities was quantified using a microscale (Mettler-Toledo, Oakland, CA, USA). A 5 mm stainless steel bead (OPS diagnostics; Diagnostics, Metuchen, NJ, USA) was added to each of the 30 mock community samples and the tissue was ground for 2 minutes at 1,200 hz on a Genogrinder 2010 (OPS Diagnostics). DNA extractions were performed using the Qiagen Puregene Kit (Qiagen, Hilden, Germany), according to the manufacturer’s protocols. Each DNA sample was quantified using a Qbit Fluorometer with the high sensitivity assay (Thermo Scientific, Waltham, USA) and diluted to a final concentration of 35 ng/μl. We performed a size selection from each DNA sample using a 0.75 X concentration of Ampure Beads XP (Beckman Coulter, Brea, CSA, USA). We retained the DNA bound to the beads as well as that in the supernatant. The bead-bound high molecular weight DNA was eluted from the beads, while we precipitated the low molecular weight fraction from the supernatant using the Qiagen Puregene Kit according to the manufacturer’s protocol. This size selection step split every sample into two exact replicates. One contained the intact high molecular weight DNA, and the other was composed of degraded low molecular weight DNA.

Each of the 60 samples was amplified using two primer combinations. One primer pair amplified the bacterial 16SrDNA (MS-27F/MS-338R [22]) at 55°C annealing temperature, while the other targeted the mitochondrial COI (mlCOIintF/Fol-degen-rev [10,23]) at 46°C annealing temperature. Both markers amplified very similar amplicon sizes of about 350 bp. The two marker types were targeted to test for differences in DNA degradation between arthropod DNA and DNA of arthropod associated microbes. PCRs were run in 10 μl volumes with ~15 ng of template DNA and 32 cycles using the Qiagen Multiplex PCR kit according to the manufacturer’s protocols. Each primer possessed a tail with conserved sequences, which served as priming sites for a second-round indexing PCR of 6 cycles. After each PCR round, samples were cleaned of residual primers by 1 X Ampure Beads XP purification. The dual indexed libraries were quantified using a Qbit as described above, pooled in equimolar amounts and sequenced on an Illumina MiSeq using V3 chemistry with 300 bp paired end reads according to the manufacturer’s protocols (Illumina, San Diego, CA, USA). The COI data was partly also used for a study on abundance estimates from sequencing data [24].

### Estimation of DNA degradation bias from Malaise traps

We evaluated DNA degradation bias under field conditions using two Malaise traps. One canopy Malaise and one ground Malaise trap were left in native rainforest of Hawaii Island (Alili Spring, Lat: 19.231°, Lon: -155.516°) for exactly two weeks in July 2015. Collecting permits were issued by the Hawaii Department of Land and Natural Resources in Honolulu. No protected species were targeted. Specimens were collected in 99% ethanol. The content of each trap was brought to the lab and stored at -20°C. The ground Malaise yielded 504 specimens and the canopy Malaise 380 specimens. The specimens were sorted into six size categories (bodylength of 0–2, 2–3, 3–4, 4–5, 5–7,7–9 mm). The size categories were based on previous estimates of body size distributions of Hawaiian arthropods and served to avoid large taxa, which are expected to contribute disproportionate amounts of DNA to a sample. DNA was extracted separately from each of the size categories. DNA extraction, size selection, PCR, library preparation and sequencing were then performed as described above for the mock communities.

### Estimation of PCR bias

Specimens were collected on Hawaii Island and identified to taxa as described above. Fifteen mock communities were created with randomized amounts of tissue for all taxa and DNA extracted and quantified from each community. We ran PCRs using the Qiagen multiplex PCR kit under different conditions targeting the mitochondrial COI gene. The PCR conditions, summarized in Table 1, included: **(1)** An exact PCR replicate of the same sample at the same cycle number (32 x) and annealing temperature (46°C), using the primer pair ARF1/Fol-degen-rev [9–10]. **(2)** A reduction of PCR cycles from 32 to 22, otherwise the same as (1). **(3)** An increase of annealing temperature by 5°C to 51°C, otherwise the same as (1). **(4)** Using the primer combination mlCOIintF/Fol-degen-rev instead of ARF1/Fol-degen-rev, otherwise the same as (1).

**Table 1:** Summary of PCR condition experiment. The table shows sample size, primer combinations, annealing temperature and PCR cycle number as well as average alpha–and beta diversity ± standard deviation. The first two rows are repeated and present an exact replicate at the same PCR conditions. Significant differences are indicated by shading of cells.

N	Primer combination	T_anneal_	# cycles	SR	Alpha div.	Beta div.
15	ARF1/Fol-degen-rev	46°C	32	39.07 ± 15.44	0.88 ± 0.05	0.05 ± 0.01
15	ARF1/Fol-degen-rev	46°C	32	40.07 ± 14.70	0.88 ± 0.04	
15	mlCOIintF/Fol-degen-rev	46°C	32	42.40 ± 15.02	0.90 ± 0.04	0.33 ± 0.08
15	ARF1/Fol-degen-rev	51°C	32	39.00 ± 14.90	0.86 ± 0.05	0.61 ± 0.14
15	ARF1/Fol-degen-rev	46°C	22	41.82 ± 16.26	0.92 ± 0.03	0.64 ± 0.09

### Sequence analysis

Reads were trimmed and assembled using PEAR [25] with a minimum quality of 30 and a minimum overlap of 50 bp. Using the FastX toolkit [26], we filtered all reads with at least 90% of the sequence at ≥ Q30 and transformed them into FASTA files. Sequences for each primer pair were trimmed off with a custom UNIX script. We performed an OTU clustering of all combined reads for each marker at a similarity cutoff of 97% using USEARCH [27]. The resulting OTU reference files for COI and 16SrDNA were compared against the NCBI database to identify the taxonomy of each sequence using BLASTn [28]. Only sequences with a significant BLAST hit were retained. COI sequences were additionally translated and all sequences containing stop codons removed as likely NUMTs [29]. Using the resulting reference libraries, we generated OTU tables for each marker using USEARCH. To account for variation in sequencing coverage, we subsampled the sequence files to 5,000 reads before OTU clustering. We calculated Simpson Index and species richness as a measure of alpha diversity for every sample using the Vegan package [30] in R [31]. As a measure of beta diversity, Bray Curtis dissimilarity between the high and low molecular weight fraction and between different PCR replicates of each sample and marker were calculated using the Ecodist package [32] in R.

Aside from community wide diversity estimates, we were also interested in the recovery of separate taxa at different storage conditions. For this reason, we compared the proportion of all recovered OTUs between high and low molecular weight fractions of our degradation experiment by linear regression. Two exact replicates at the same PCR conditions served as a baseline for PCR stochasticity.

## Results

Sequences were of high quality and good coverage for most samples. After quality filtering, assembly and primer trimming, we recovered, on average, 15,553 16SrDNA and 15,444 COI sequences for the degradation experiment, 8,225 16SrDNA and 7,462 COI sequences from the Malaise traps and 25,400 COI sequences for the PCR condition experiment. Three out of 60 COI samples and 16SrDNA samples of the DNA degradation experiment, one out of 24 COI samples and 16SrDNA samples of the Malaise trap experiment and five out of 75 samples of the PCR condition experiment, were removed from the analysis due to low coverage.

### Alpha diversity

**Fig 1:**
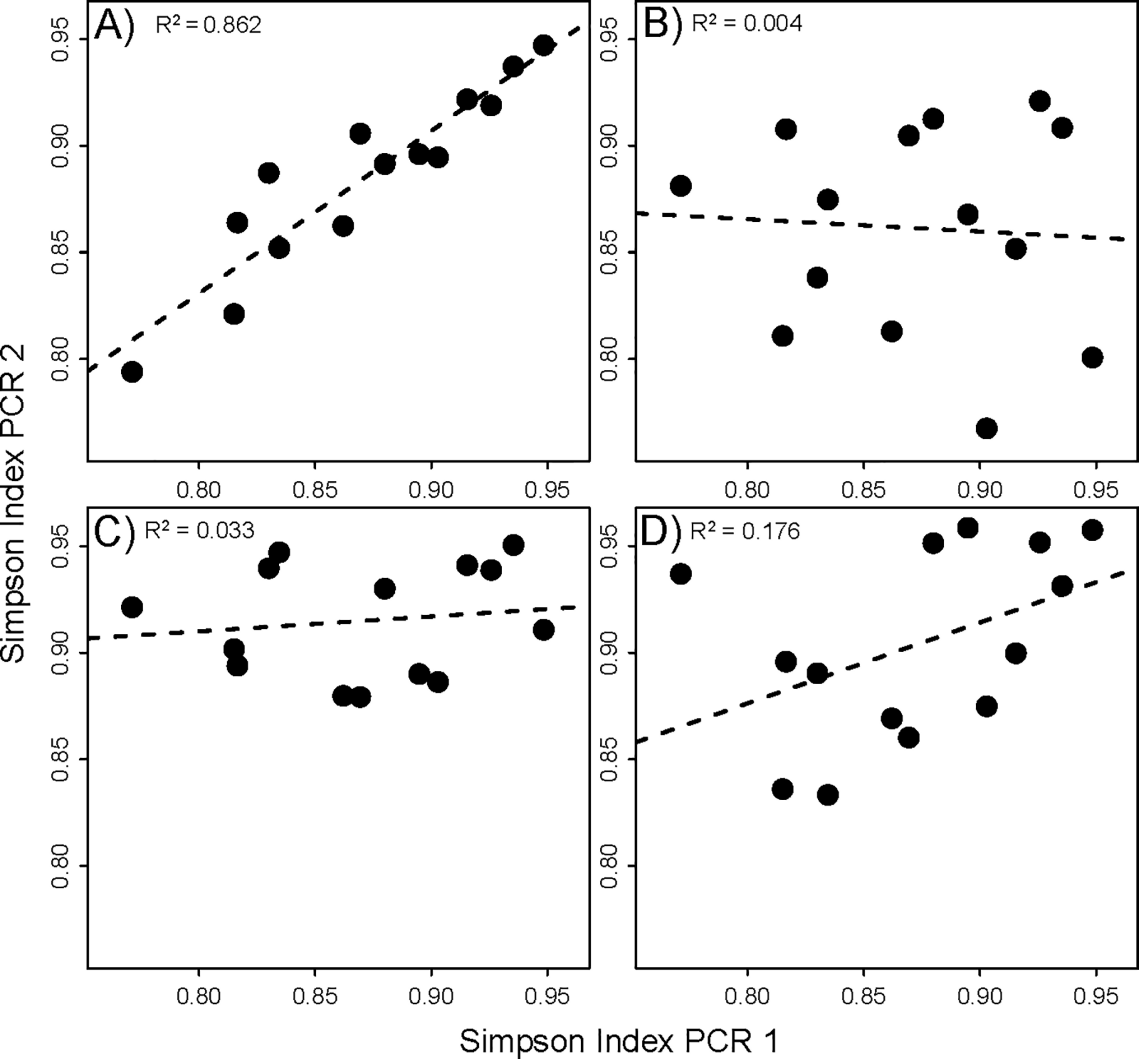
Association of Simpson indices for PCR replicates of the same samples. The X-axes represent the Simpson indices for the same sample in all cases, amplified with 32 cycles and a 46°C annealing temperature. The Y-axis corresponds to varying changes of PCR conditions: **A)** No change, exact PCR replicate at 46°C, **B)** a reduction of PCR cycle number by 10, **C)** an increase of the annealing temperature by 5°C, **D)** use of a different forward primer. Dashed lines represent linear regression lines.

Variation of PCR conditions did not recover significantly different average alpha diversities between samples (Pairwise Wilcox test, *P* > 0.05). This holds true for species richness as well as Simpson indices (Figure A in S1 File, Table 1). However, very pronounced differences were found for an association of alpha diversity of PCR replicates at different PCR conditions (Fig 1A–1D). An exact replicate at the same PCR conditions resulted in a very good correlation of alpha diversity for the two samples (*R*^*2*^ *= 0*.*862*, *P < 0*.*05*). A change of annealing temperature and a reduction of PCR cycle numbers led to vastly different diversities, with no significant correlation between diversity of sample replicates (*R*^*2*^ *< 0*.*05*, *P > 0*.*05*). The use of a different forward primer during PCR also resulted in a considerable reduction of the coefficient of determination between the sample replicates (*R*^*2*^ *= 0*.*176*, *P < 0*.*05*). Thus, even though the average alpha diversity across all samples was not significantly different between conditions, changing PCR conditions had a significant effect on the diversity of separate samples (Table 1).

**Fig 2:**
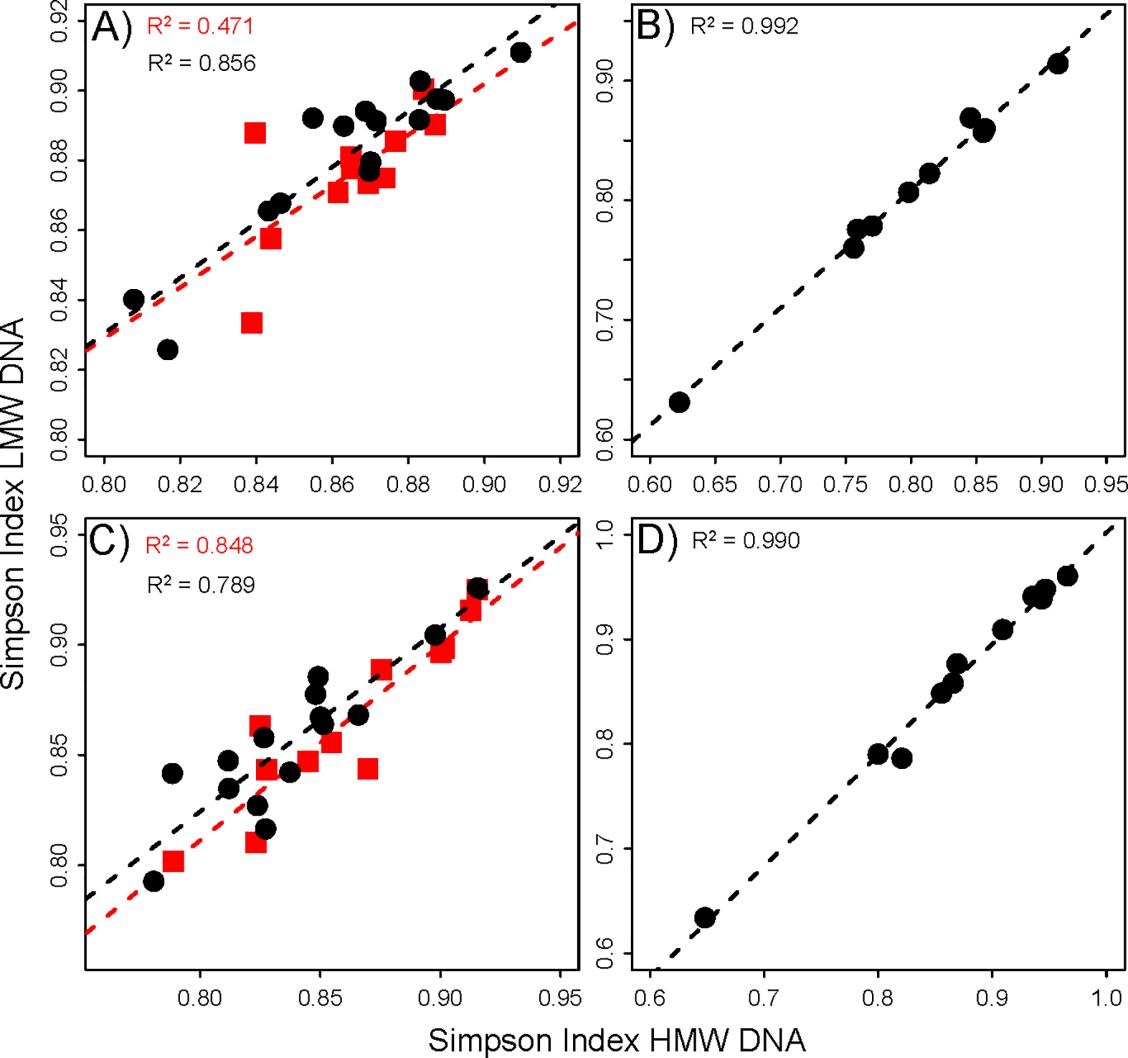
Simpson indices and their associations for the DNA degradation experiment. **A-D)** Association of Shannon indexes between high and low molecular weight DNA fraction for exact replicates of the same sample for **A)** samples stored in the freezer (red squares) or stored at room temperature (black circles) and amplified for COI **B)** Samples from actual Malaise traps amplified for COI. **C)** samples stored in the freezer (red squares) or stored at room temperature (black circles) and amplified for bacterial 16SrDNA. **D)** Samples from actual Malaise traps amplified for microbial 16SrDNA. The X-axis represents the high molecular weight and the Y-axis the low molecular weight fraction. Dashed lines represent linear regression lines.

We recovered considerably more microbial than arthropod OTUs in our degradation experiment and in the Malaise trap samples (Table 2). No significant difference of average alpha diversity between high and low molecular weight DNA fraction was discovered for any of our DNA degradation experiments. We also did not find significant differences of alpha diversity between samples stored in the freezer and those kept at room temperature (Figure B in S1 File, Fig 2A; Table 2). However, Malaise trap samples showed a considerably lower species richness than our degradation experiments (Table 2, Figure B in S1 File).

**Table 2:** Summary of DNA degradation experiment. The table shows amplified marker, sample size, primer combinations, DNA integrity (high vs. low molecular weight), storage conditions of samples, as well as average alpha (species richness & Simpson index)–and beta diversity ± standard deviation.

Gene	N	DNA integr.	Storage	SR	Alpha div.	Beta div.
COI	15	High	Freezer	26.36 ± 6.92	0.86 ± 0.02	0.09 ± 0.05
COI	15	Low	Freezer	28.09 ± 7.05	0.88 ± 0.02	
COI	15	High	Room temp.	30.60 ± 3.48	0.87 ± 0.03	0.12 ± 0.04
COI	15	Low	Room temp.	32.20 ± 3.745	0.88 ± 0.02	
COI	12	High	Malaise trap	18.91 ± 7.31	0.80 ± 0.08	0.05 ± 0.02
COI	12	Low	Malaise trap	19.455 ± 7.44	0.81 ± 0.08	
16S	15	High	Freezer	110.00 ± 42.85	0.86 ± 0.04	0.09 ± 0.03
16S	15	Low	Freezer	111.67 ± 39.29	0.87 ± 0.04	
16S	15	High	Room temp.	122.47 ± 37.93	0.84 ± 0.04	0.11 ± 0.03
16S	15	Low	Room temp.	125.80 ± 39.11	0.86 ± 0.03	
16S	12	High	Malaise trap	61.18 ± 35.22	0.87 ± 0.09	0.07 ± 0.03
16S	12	Low	Malaise trap	56.82 ± 35.03	0.86 ± 0.10	

We found a fairly good association between alpha diversity for the high and low molecular weight fractions of the same samples in our degradation experiment (0.471 ≤ R^2^ ≥ 0.856, *P* < 0.05). However, compared to simple PCR replicates, the association of alpha diversities for high and low molecular weight DNA was considerably worse. No pronounced effect of freezer storage or room temperature storage on the association of alpha diversities for high and low molecular weight samples was found. This holds true for mitochondrial COI and microbial 16SrDNA markers alike (Fig 2A & 2C). Interestingly, the best association of alpha diversity between high and low molecular weight DNA fractions was found for actual Malaise trap samples (Fig 2B & 2D). The Simpson indices for high and low molecular weight DNA of both mitochondrial COI and microbial 16SrDNA showed a near perfect association for the Malaise trap samples (R^2^ > 0.99, *P* < 0.05).

### Beta diversity

**Fig 3:**
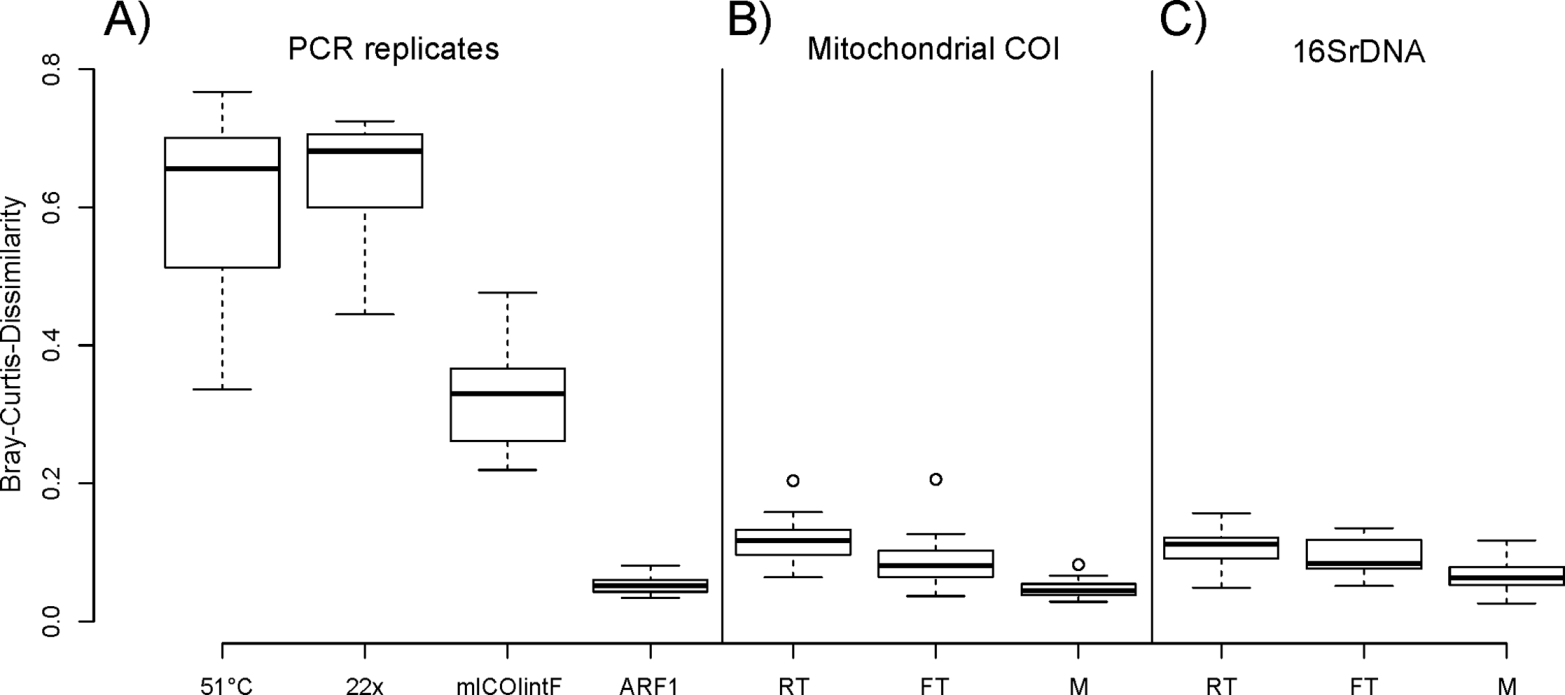
Bray Curtis dissimilarity between PCR replicates and between high and low molecular weight DNA fractions in the degradation experiment and from Malaise trap samples. **A)** Bray Curtis dissimilarity between PCR replicates of the same mock community samples at 5°C different annealing temperatures (51°C from 46°C), with a reduced PCR cycle number (22 x from 32 x), using a different forward primer (mlCOIintF instead of ARF1), and for two exact replicates under identical conditions (to test for stochastic PCR bias). **B)** Bray Curtis dissimilarity between high and low molecular weight DNA fractions for community samples stored under freezer (FT) and room temperature (RT) conditions or from an actual Malaise trap (M) and amplified for mitochondrial COI and **C)** for microbial 16SrDNA.

Exact PCR replicates of the same sample under identical annealing temperatures resulted in a very low Bray Curtis dissimilarity between the two replicates (Fig 3A; Table 1). Simple PCR stochasticity thus introduced only a small bias into our analyses. Nevertheless, the distance was significantly different from zero (*one-sample t-test*, *t = 15*, *P < 0*.*05*). In contrast, an increase of annealing temperature by 5°C, or a reduction of PCR cycles by 10, led to vastly different community compositions and thus a significantly increased beta diversity. A less pronounced, albeit significant, increase of Bray Curtis dissimilarity was found for PCR replicates using two different COI primers (*Pairwise Wilcoxon test*, *FDR-corrected P-value < 0*.*05*; Fig 3A; Table 1).

A comparison of the recovered communities from high and low molecular weight DNA fractions did not show a significantly higher Bray Curtis dissimilarity than simple PCR replicates (Fig 3B & 3C; Table 2). The recovered beta diversity was very low for all degradation experiments (Fig 3A–3C; Tables 1 & 2). The recovered Bray Curtis dissimilarity between samples stored at room temperature and freezer conditions was not significantly different (*Pairwise Wilcoxon Test*, *FDR-corrected P-value > 0*.*05*). However, we found a trend for lower beta diversity between high and low molecular weight DNA from samples that were consistently frozen, over those stored at room temperature (Fig 3B & 3C). The lowest Bray Curtis dissimilarity was found for actual Malaise trap samples, for mitochondrial COI as well as microbial 16SrDNA (Fig 3B & 3C). In fact, the Bray Curtis dissimilarity between high and low molecular weight DNA from Malaise trap samples was not distinguishable from simple PCR replicates.

### Recovery of different taxa

**Fig 4:**
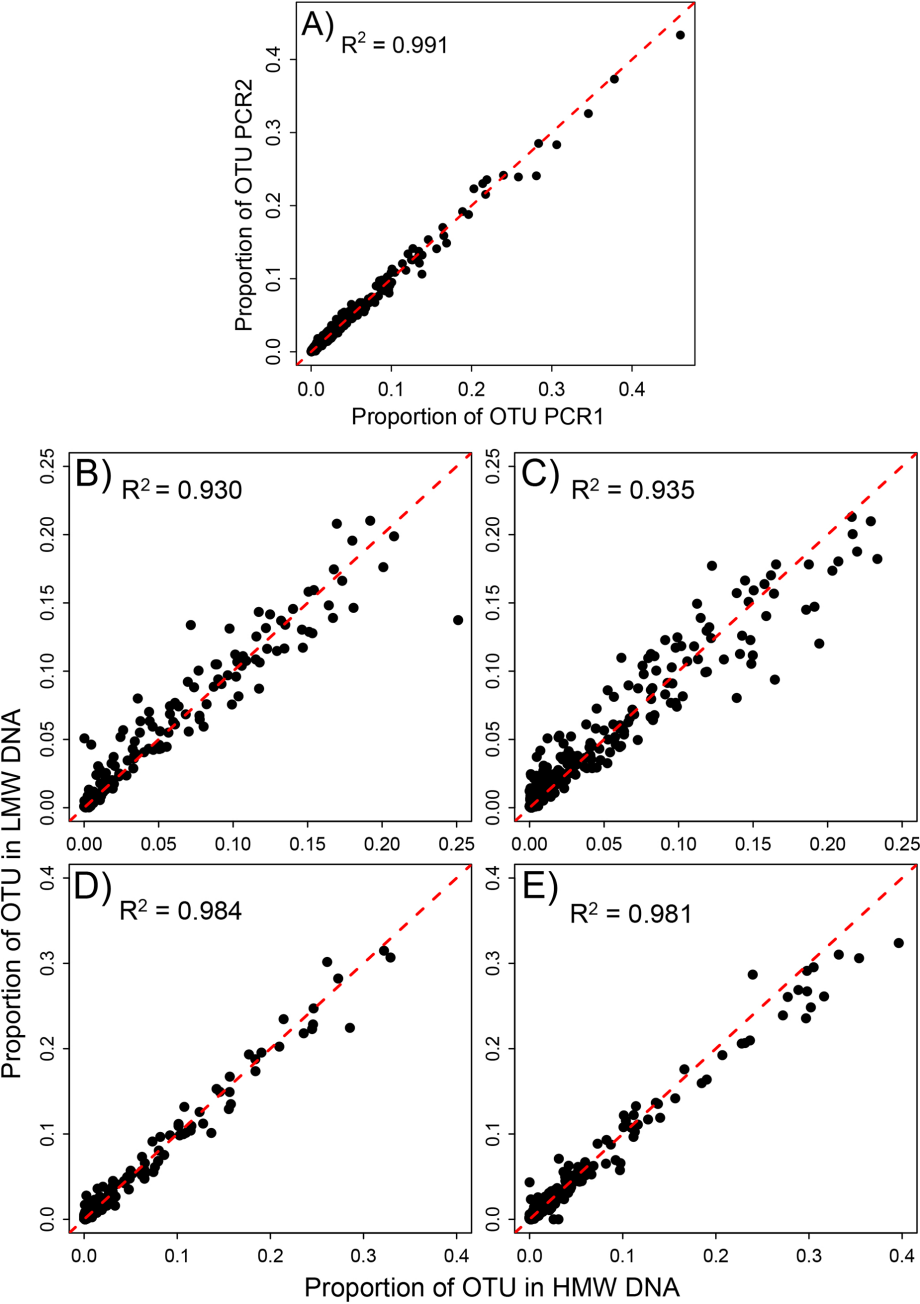
Associations of OTU read proportions between exact PCR replicates and between high and low molecular weight DNA fractions. **A)** Relative OTU abundances for two exact replicates at the same PCR conditions. **B)** For mitochondrial COI amplified from samples stored at -20°C and **C)** at room temperature. **D)** For microbial 16SrDNA amplified from samples stored at -20°C and **E)** at room temperature. The dashed line represents the 1:1 line.

OTU proportions between samples that were amplified at exactly the same PCR conditions showed a very narrow association (*R*^*2*^ *= 0*.*991*, *P < 0*.*05*). The proportions of OTUs among the total read populations thus were almost identical between exact PCR replicates (Fig 4A). We also found a good association of OTU proportions between high and low molecular weight DNA fractions in our degradation experiment (Fig 4B–4E). However, compared to PCR replicates, a much wider distribution of datapoints around the 1:1 line was evident for the degradation experiment. No pronounced differences in the association of recovered OTU proportions between high and low molecular weight DNA were found for samples kept frozen or those stored at room temperature. This holds true for mitochondrial COI (R^2^_FT_ = 0.930, R^2^_RT_ = 0.935), as well as microbial 16SrDNA (R^2^_FT_ = 0.984, R^2^_RT_ = 0.981). However, a wider spread of data points around the 1:1 line became evident for samples stored at room temperature (Fig 4B & 4C).

Both high and low molecular weight DNA fractions in our degradation experiment recovered most of the input taxa. In total, only eight specimens in four species were not recovered by sequencing (1 Diptera, 3 Hymenoptera and 4 Isopoda). As the primers that we used here have already been shown to be less efficient in amplifying isopods and hymenopterans [24], this is not surprising. Two additional spider specimens were only recovered in the low molecular weight fraction. For most taxa, the proportion found in the low and high molecular weight DNA fractions was very comparable. The median fold change between taxon proportions in the low and high molecular weight fraction was higher in the samples stored at room temperature (FC = -0.093) than in those stored in the freezer (FC = -0.019) (Figure C in S1 File), albeit not significantly different. The specimens for which we find a considerable change of abundance (> 2-fold) between high and low molecular weight fraction were dominated by spiders (16/26) and the Collembola genus *Homidia* (6/26) (Figure D in S1 File).

## Discussion

The marginal bias in alpha and beta diversity between exact PCR replicates suggests that it may not always be necessary to perform PCR replicates of the same sample in metabarcoding studies. Differences between two replicate PCRs primarily concerned the recovery of rare sequences. However, even slight variation in PCR conditions had a profound effect on diversity estimates. Primer choice, annealing temperature and PCR cycle number all led to the recovery of vastly different communities. It is therefore important to use standardized conditions in metabarcoding analyses to achieve comparable results across study systems and laboratories. The pronounced bias found for PCR replicates using two different primer pairs also suggests that degradation bias cannot reliably be scored using differently sized PCR amplicons. Any signature of DNA degradation would likely be swamped by differential amplification. The size selection protocol we used here is a straightforward and simple alternative to differently sized amplicons. But it needs to be noted that it does not directly estimate which taxa are lost from a community sequence analysis due to degradation. It only quantifies whether taxa are over or underrepresented among different DNA size classes, e.g. the DNA of which taxa degraded faster or slower than that of others. Only those parts of the low molecular weight fraction are amplified, which are still in the size range of the primers used. It would thus be advisable to target very short amplicons to estimate degradation bias. Alternatively, PCR free libraries with different insert sizes could be used to quantify degradation bias. However, compared to amplicon sequencing of size selected samples, this significantly increases the cost and effort.

Our results suggest that differential DNA degradation can contribute to biased taxon recovery rates. But in contrast to amplification bias, this DNA degradation bias appears of minor importance. Storage temperature conditions had a detectable, but only small effect on the average alpha or beta diversity or taxon recovery. This finding suggests that it is not always necessary to transfer samples to freezer conditions right after sampling in order to perform meaningful metabarcoding analyses. Especially during sampling expeditions in remote places, this is usually not possible. For Illumina sequencing, short sequence stretches of multi copy loci are amplified, further mitigating the importance of DNA degradation bias. In light of these results, passive trapping methods, like Malaise traps, appear well suited for metabarcoding applications, even after certain periods of suboptimal storage. Surprisingly, community samples from actual Malaise traps even showed a slightly lower degradation bias than our freezer stored mock communities. The Malaise traps were left in the field for only two weeks, while our degradation experiment exposed specimens to four weeks of unfavorable storage, before freezing them. This may be a major reason for the observed differences. Moreover, our Malaise trap samples contained a considerably lower number of species, compared to the mock communities. The increased complexity of our mock communities might lead to a stronger effect of degradation bias. In this regard, it is noteworthy that degradation bias in our experiments was particularly pronounced for two taxa, a spider and a springtail species. Spiders are indeed known for their fast DNA degradation [19], possibly due to very efficient DNAses in their digestive fluids. Our Malaise traps contained almost no Araneae or Collembola. Degradation bias might thus be dependent on the species analyzed, and our results might not be representative for all study systems.

It should be noted, that the DNA degradation was limited to relatively short periods of time in our study (e.g. 2–4 weeks). Longer term storage at suboptimal conditions, e.g. in a museum collection, may induce more pronounced DNA degradation. DNA degrades exponentially [33], hence much larger effects of degradation bias may be caused, when rapid degradation sets in. Recent developments in technology have led to considerable increases of the length of barcode sequences, which can be routinely targeted by next generation sequencing [34]. Such long read approaches may also soon be adapted to metabarcoding applications. Sequencing of long amplicons, may be much more susceptible to degradation bias, urging for careful analysis of communities based on long read sequences. Our results show that passively collected samples may be well suited for the analysis of the microbiota of arthropods. Even after long term storage, we recovered very comparable microbial communities between degraded and intact DNA fractions. However, other work suggests that storage conditions can affect microbial community analyses [20]. Also, by targeting all microbes associated with an arthropod community, we used a coarse approach. More pronounced differences in microbial communities of separate taxa, or tissues within a taxon may be present. The utility of samples may be condition dependent, and it is advisable to test the effect of degradation bias before embarking on large scale metabarcoding analysis from passively collected arthropods and their microbiota.

## Conclusions

DNA degradation bias only had a small effect on the recovery of diversity by arthropod community metabarcoding. Variation of PCR conditions emerged as a more severe biasing factor. Our results suggest that the Arthropod macrobiome of a given location and its associated microbiota can be scored by sequencing passively collected samples.

## Supporting information

S1 File File containing Supplementary Figures A-D. (DOCX)
