# Supplementary material for: The effect of DNA degradation bias in passive sampling devices on metabarcoding studies of arthropod communities and their associated microbiota
Source: PLoS One. 2018 Jan 5;13(1):e0189188. doi: 10.1371/journal.pone.0189188 (PMC5755739; doi:10.1371/journal.pone.0189188)
Supplement: S1 File — (DOCX) [file pone.0189188.s001.docx]

**Supplementary Figures**


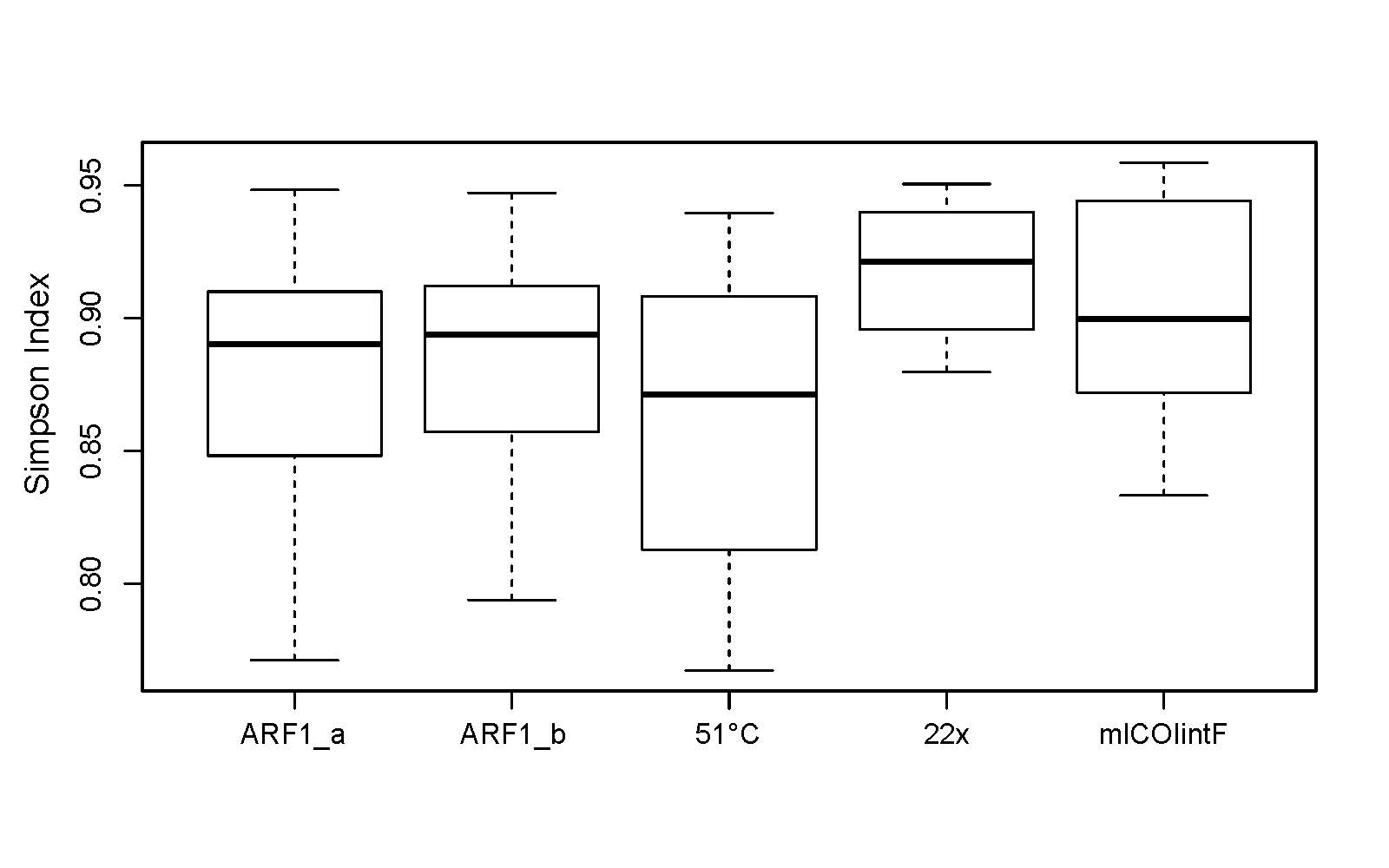


**Fig.** **A** Simpson indices based on mitochondrial COI of our PCR replicate experiment. For exact replicates at the same PCR conditions (ARF_a & ARF_b), after an increase of the annealing temperature by 5°C (51°C), after reduction of the PCR cycle number by 10 (22X) and after using a different forward primer (mICOIintF).


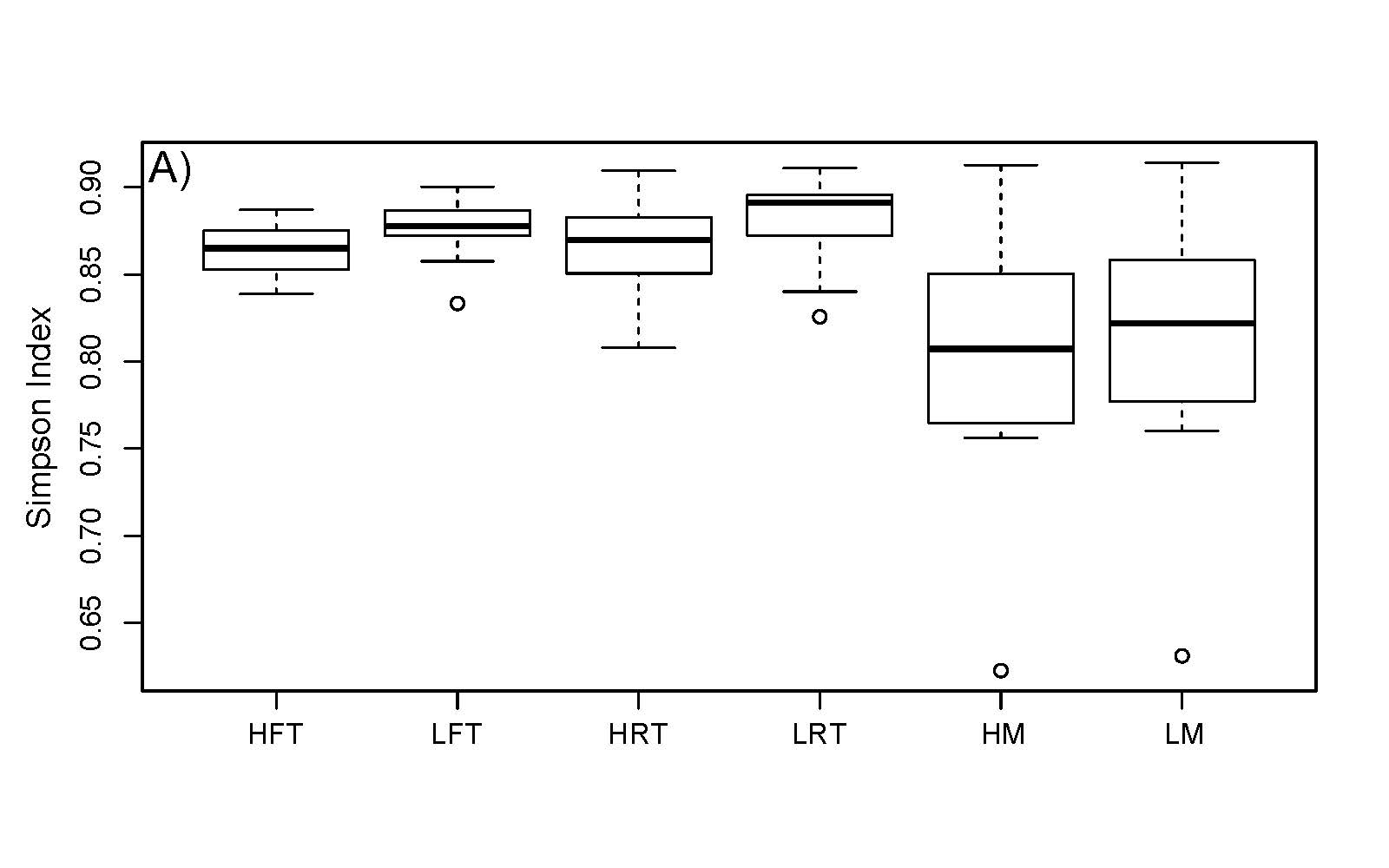

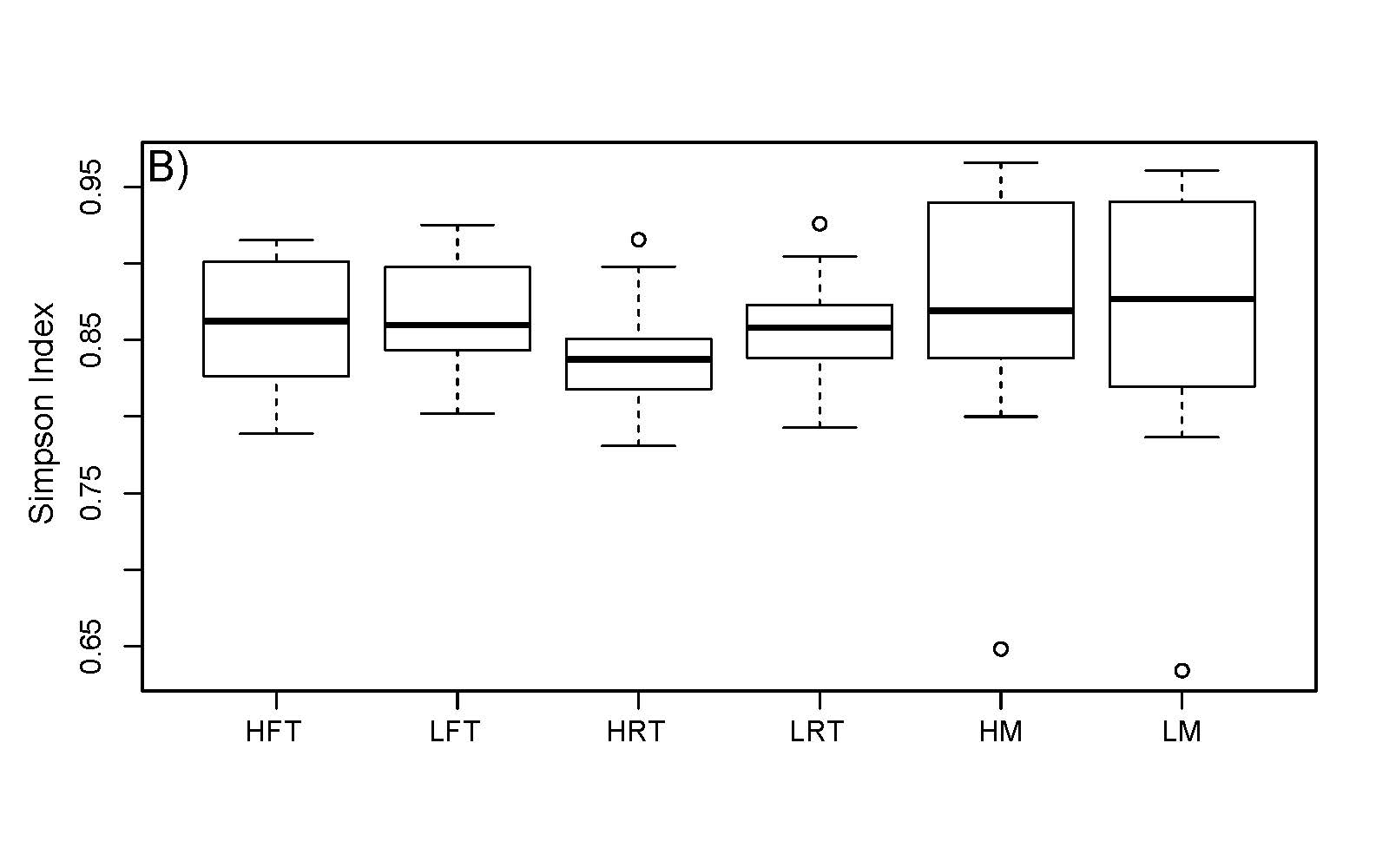


**Fig.** **B** Simpson indices for high (H) and low (L) molecular weight DNA for all community samples of our degradation experiment (FT = freezer temperature, RT = room temperature) and the Malaise trap samples (M). Plot **A)** shows the result for arthropod mitochondrial COI and **B)** for microbial 16SrDNA.


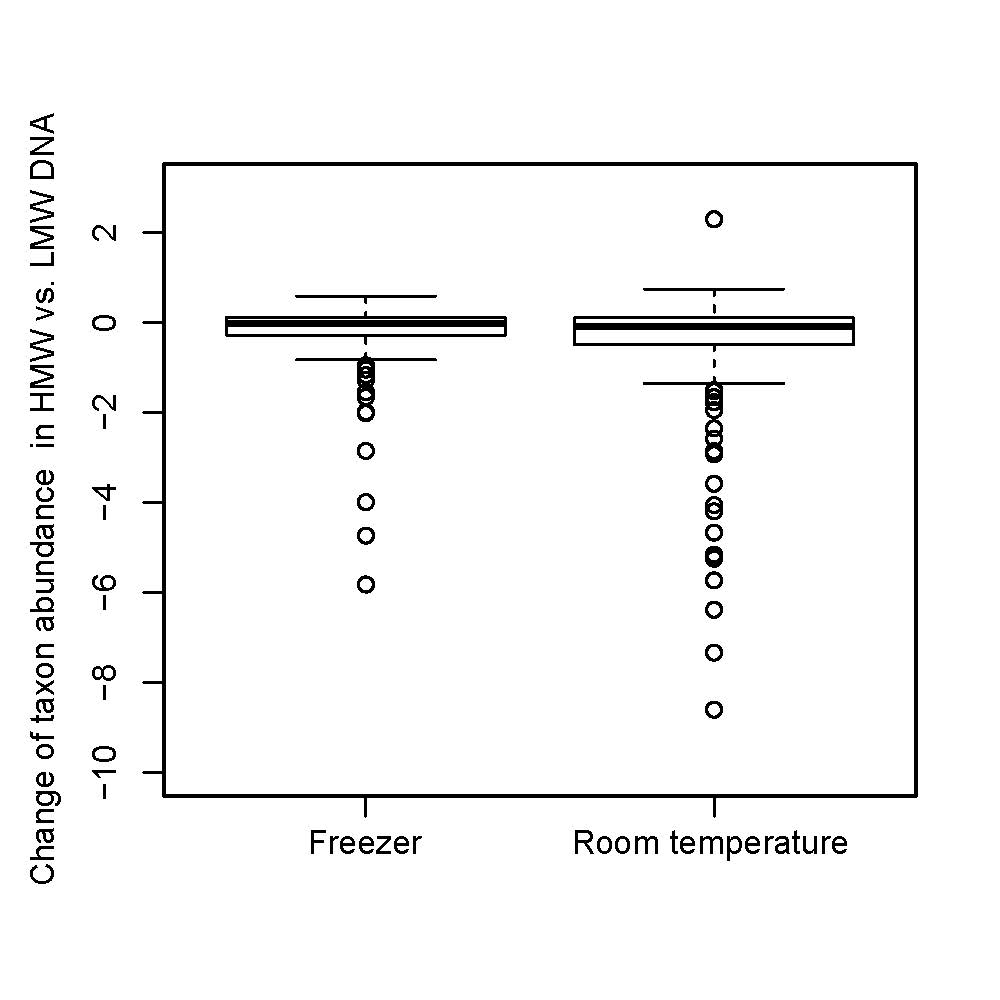


**Fig.** **C** Fold change of taxon abundance between high and low molecular weight samples of our degradation experiment and for samples stored at freezer and room temperature conditions. Outliers below -10 are not shown for reasons of clarity. A fold change of zero corresponds to identical abundances of a taxon’s reads in high and low molecular weight DNA.

**Fig. D** Number of samples for taxa for which we found a more than 2 -fold difference of read abundance between high molecular weight and low molecular weight DNA fraction in the degradation experiment. The jumping spider Havaika sp. and the springtail Homidia sp. dominate these taxa.
